# A qualitative exploration of stakeholder perspectives on the implementation of a whole school approach to mental health and emotional well-being in Wales

**DOI:** 10.1093/her/cyad002

**Published:** 2023-01-30

**Authors:** R Brown, J Van Godwin, A Edwards, M Burdon, G Moore

**Affiliations:** DECIPHer, Cardiff University, SPARC, Maindy Road, Cardiff, Wales CF24 4HQ, UK; Wolfson Centre for Young People’s Mental Health, Cardiff University, SPARC, Maindy Road, Cardiff, Wales CF24 4HQ, UK; DECIPHer, Cardiff University, SPARC, Maindy Road, Cardiff, Wales CF24 4HQ, UK; DECIPHer, Cardiff University, SPARC, Maindy Road, Cardiff, Wales CF24 4HQ, UK; DECIPHer, Cardiff University, SPARC, Maindy Road, Cardiff, Wales CF24 4HQ, UK; DECIPHer, Cardiff University, SPARC, Maindy Road, Cardiff, Wales CF24 4HQ, UK; Wolfson Centre for Young People’s Mental Health, Cardiff University, SPARC, Maindy Road, Cardiff, Wales CF24 4HQ, UK

## Abstract

Early intervention to support mental health and well-being of school-aged children may be of significant benefit in preventing escalation of mental health problems in later life. While there are limitations to current understanding of the best ways for schools to support mental well-being, a whole school approach (WSA), involving all those who are part of the school system in creating and sustaining a supportive environment where health is prioritized, may be effective. This research explored stakeholder views of this approach, as part of a contract commissioned by the Welsh Government to conduct an evaluability assessment of a WSA. Semistructured focus groups and interviews were completed with stakeholders from the health and education sectors, as well as parents, to explore how a WSA may operate in a Welsh context and barriers and facilitators to potential implementation and outcomes. Findings suggest that existing pressures on schools may impact implementation of a WSA, with school staff already time poor and many staff experiencing their own mental well-being challenges. Implementation may be supported by clear guidance at local and national levels, funding for staff time and training and stakeholder involvement at all stages. Long-term monitoring and evaluation are also needed to understand system changes.

## Introduction

Data collected prior to the COVID-19 pandemic suggest that, in Wales, over 60% of children and young people self-report feeling challenges to their mental health weekly, including low mood, anxiety and feeling fearful [1]. While experience of negative emotions and reactions is a normal part of everyday life [2], repeated and sustained mental health challenges in childhood may have longer-term impacts, including worsening educational outcomes [3] and increased likelihood of problems with mental health in later life [4]. Data show a general deterioration in youth mental health prior to the pandemic, with increases in a range of mental health conditions among people younger than 18 years old since 2004 [5]. Since the easing of lockdown restrictions, impacts on mental health are still emerging but do not indicate uniform deterioration. While evidence suggests overall growth in challenges to young people during the pandemic [6], around a quarter of young people suggest an improvement in their mental well-being during the first UK lockdown [7]. However, those already experiencing mental health challenges and those from most socio-economically deprived groups report worsening mental health during this period [7], suggesting a potential increase in inequity through exacerbating existing vulnerabilities.

Evidence suggests that early intervention to support mental well-being may be of significant benefit in school-aged populations [8]. There has been an increased focus on school-based mental health support in Wales in recent years, e.g. in the requirement for all schools to make school counselling available to pupils from year 6 onwards (aged 10–18 years) [9]. However, over a quarter of young people still feel that school-based help for mental health challenges is absent [10].

While schools can act as providers of formal support for mental health, such as counselling, they may also be sites of broader influence. While there is an absence of high-quality evidence on how schools may most effectively support mental and emotional health [11], evidence does suggest that where staff–student relationships and sense of school connectedness are higher, pupils report lower rates of mental health problems [12]. Furthermore, where pupils perceive that their school prioritizes health and well-being, both for pupils and staff, this is seen as supportive of better overall well-being [3]. Poorer staff well-being is associated with poorer pupil well-being [13], illustrating the importance of a broader whole school focus in supporting improved outcomes. Even prior to the added pressures created by the COVID-19 pandemic, a quarter of teachers in Wales reported current or historic experience of mental health issues [14]. More recent data from across the United Kingdom suggest that this may have risen to over 60% in 2021 [15].

In 2018, the Welsh Government committed to the adoption of a whole school approach (WSA) to mental health and well-being, culminating in the 2021 ‘Framework on Embedding a Whole School Approach to Emotional and Mental Well-Being’ [16]. Through this, it is now a statutory requirement for all educational settings to work towards taking a WSA within their everyday practices to support mental health and emotional well-being. While the framework notes that schools should all aim to integrate a WSA into the curriculum and use the document as a tool to review their own progress, flexibility of delivery and school-specific practices to support mental health are encouraged, allowing for variation within WSAs utilized. Additional to this, a new curriculum for Wales has been developed, which, from 2022, includes health and well-being as an Area of Learning and Experience on par with all other curriculum subject areas for the first time (see https://hwb.gov.wales/curriculum-for-wales/health-and-well-being/). Together, these indicate a strengthened focus on well-being within the education system in Wales.

A WSA aims to involve all those who are part of the school system in creating and sustaining a supportive environment where health is prioritized [17]. It involves co-ordinated working, both within schools and with supporting external parties, to develop and implement data-driven practices reflecting the needs and resources of each school [18]. While there is growing evidence of positive effects of WSAs in areas such as social and emotional adjustment [19, 20], there are limitations to current understanding of school structures and actions necessary for effective delivery and programme sustainability [21]. Evidence suggests that insufficient focus on implementation processes is likely to undermine effectiveness [22], meaning that the identification of potential barriers and facilitators to implementation is important.

This research was carried out during the development of—but independent to—the Framework on Embedding a Whole School Approach to Emotional and Mental Well-Being, as part of a contract commissioned by the Welsh Government to conduct an evaluability assessment of the WSA to mental health. Data collection was completed prior to its issuance to schools in March 2021, but the final report was published after this date (see Whole School Approach to Mental and Emotional Wellbeing: evaluability assessment | GOV.WALES). It explored perceptions of WSAs in general, thoughts on potential implementation in Wales and future impacts and the likely barriers and facilitators to adoption of a WSA. Participants were key stakeholders within and around educational settings in Wales.

## Materials and methods

### Sampling and recruitment

Semistructured interviews and focus groups were completed with a range of key stakeholders involved in schools and/or mental health service provision in Wales. Semistructured approaches focus on the knowledge and experience of participants [23], here with emphasis on perceptions of current actions on mental health and emotional well-being within schools, defining a WSA, how a WSA to mental health and emotional well-being may be implemented and what outcomes it may lead to.

Interviewees were identified through existing networks and through discussions with the Welsh Government, informed by the needs of the commissioned evaluability assessment of the WSA to mental health. Participants were drawn from across Wales, including staff from local authorities, mainstream and non-mainstream school staff and governors, regional school consortia, school inspectorates, specialist mental health services and parents. Sampling was constrained by the study budget and timescale (as a commissioned study), meaning that saturation was not aimed for, but sampling was informed by the likelihood of key insights from those approached. Overall, 28 people were interviewed (26 across five groups and two individual interviewees who were unable to attend group sessions).

**Table:** 

Group 1	Group 2	Group 3	Group 4	Group 5
*N* = 6	*N* = 4	*N* = 5	*N* = 5	*N* = 6

Sessions all lasted for 60–90 minutes. Sociodemographic data on participants were not collected as they were not a feature of recruitment.

### Data collection and analysis

Ethics approval was granted by the Research Ethics Committee, School of Social Sciences, Cardiff University, with processes guided by Social Research Association guidelines on good practice in reducing potential harms to participants. Participants were provided with bilingual (Welsh/English) consent and information sheets prior to confirming their interest in taking part. They were invited to ask questions and were informed of their right to withdraw prior to full report publication. All interviews were conducted in English.

Data collection took place between December 2020 and June 2021, when COVID-19 restrictions were variably operating and impacting permitted gatherings and locations of work. As such, all interviews were conducted online using Microsoft Teams in line with the university’s risk assessment processes. Some school staff were on-site at the time of interview as schools were providing on-site teaching for a limited number of pupils.

Online interviewing has been noted as convenient, accessible and time-saving by research participants [24]. Here, it facilitated attendance by participants across Wales who may otherwise have been geographically restricted if asked to convene in one location. All discussions were audio-recorded, with both written and verbal consent from interviewees. Discussions were based on a predesigned topic guide that allowed focus on main study aims but with flexibility to explore new areas if they emerged.

All interviews were transcribed and then analysed thematically, to identify, analyse and report patterns [25]. This approach reflects the focus on meaning and richness in the data [23]. Transcripts were initially read openly and repeatedly by the lead author to develop an initial coding framework, incorporating study aims and also unplanned themes. This was discussed and refined within the research team to consider the views of others involved in interviewing. About 20% of transcripts were then second-coded by another team member, with the aim of exploring areas of disagreement and increasing consistency of the analysis [26]. This led to further refinement of the coding framework, which was then applied for final theme development.

## Results

This section presents findings from analysis of interviews with key stakeholders. It considers how a WSA was understood by participants, including what it is and what the programme aims are. It focuses on participant views of what may constitute core actions for schools and other delivery partners in the implementation of a WSA, including potential barriers that may act to impede these actions. Quotes are used throughout as illustrative examples of the theme being discussed.

### Defining the WSA

Participants were asked to consider what is meant by a WSA in relation to mental health and emotional well-being. There was widespread agreement among interviewees about what constitutes a WSA, with the approach characterized as being about system-wide changes (both short and long term) in mental health support, inclusivity and joined-up working:

All parts of the school, working together and being committed and it (WSA) should be embedded in the policy and the practice and the overall ethos of the school. (G2, P5)

A WSA was seen as about both prevention through mental health and well-being education and prevention of escalation through early intervention and support for those with more complex needs. It was seen as involving all within and around the school system, including pupils, school staff, families and external agencies who may provide expertise and support. While pupils were noted as a focus of the approach, it was argued that the well-being of school staff was also fundamental and should be a core element of a WSA:

There has to be a focus on staff well-being as well…you can’t pour from an empty cup, or you have to fit your own oxygen mask first. We have to be focussing on whether staff within the mainstream or within the whole school setting are in a position to support pupils themselves. (G2, P3)

It was further noted that a WSA should be underpinned by a shared ethos in relation to mental health. While it was acknowledged that ‘ethos’ is difficult to define and to recognize when it exists, it was noted as very important, as both a feature of a WSA and an outcome. This ethos was typically described as including a shared supportive language and schools being seen as safe places to discuss mental health. This involves system-wide changes distinct from more reactive, intervention-based, approaches:

It’s not about big interventions, and you know, standalone interventions, it’s about the whole school isn‘t it, and how somebody’s welcomed when they come through the door, the language, you know, it’s everything isn’t it? It’s about the way the school looks, how welcoming it is, it’s everything within that setting. (G3, P2)

### The aims of a WSA

The aims of a WSA highlighted by participants included the following: embedding a positive and open culture around mental health and emotional well-being in the everyday practices of the school; inclusivity and involvement of all those within the system in development and delivery; effective working with external partners, including mental health specialists, and delivery of universal and more targeted provision. Many stressed the long-term nature of this process, noting that aims of measurable improvements to mental health and emotional well-being are likely to be dependent on initial implementation activities and the effectiveness of embedding a WSA within the school system.

There was extensive discussion about the varying nature of schools in Wales, between primary and secondary settings, rural and urban schools, mainstream and non-mainstream provision and Welsh-medium schools, with acknowledgement that a WSA was likely to—and should be allowed to—vary according to the setting. However, some shared understanding of aims at a national level was valued as providing clarity and facilitating consistent practice.

### Core actions for schools and partners to undertake for WSA implementation

Participants were asked what steps they felt were fundamental to WSA implementation. Identified themes included stakeholder involvement, review of existing practice and understanding the needs of the school population.

#### Stakeholder involvement

Stakeholder consultation, including school staff, pupils and families, was viewed as essential in the implementation stages of a WSA, to secure buy-in to potential new activities and to ensure a shared understanding of the rationale for them. Some suggested—where staff size allowed—the appointment of a ‘champion’ to co-ordinate initial activities, as long as that champion had sufficient authority within the school to compel action. However, it was emphasized that no single person should be expected to deliver a WSA:

The whole school approach, I think as much as it’s driven by the leadership team, I don’t think it’s one person’s responsibility no matter where they are in that hierarchy within the school. (G2, P1)

Pupil consultation, either through existing structures such as school councils or through newly convened groups, was also highly valued, along with family engagement. However, it was noted that both these can be challenging, with significant variations between primary and secondary settings:

Family involvement is – it is obviously really massive – but a real challenge. Something that’s easier to achieve, probably with primary age pupils because there’s parents they have more to do with the school because the pupils younger whereas often secondary don’t, and that’s the biggest challenge I’d say for secondary schools. (P1)

#### Review of existing practice

Most felt that schools were frequently engaged in activities already that were either part of, or supportive of, a WSA and that it was therefore key to review what was already in place. This included review of existing policies, mapping assets and identifying existing practices on mental health and emotional well-being. This should be an inclusive process involving those across the school system rather than focusing only on those in leadership positions in order to get a more accurate reflection of practice:

If you were asking a school to map what they do, or we are asking them to fill in this assessment tool, then it needs to be emphasized that it’s the correct person…it needs lots of different people in the school, because you know, I managed to get into primary school once, and meeting the school’s coordinator and her opinion and vision of the school was completely different from the new person that I met. (G3, P2)

For policy review, it was suggested that the content of existing documentation on health and well-being was assessed for ‘fit’ with the aims and ethos of the WSA, but that this was insufficient for a genuine system-wide change. Policies on behaviour management and disciplinary practices, safeguarding and staff well-being should also be reviewed due to risk of inconsistencies in ways of working:

An example of where it’s really poor is when you’ve got a behaviour department and you’ve got a well-being department and it’s completely separate and nobody knows where the child actually sits…so you get a child who is having a panic attack or something and they’re in trouble because they’re also late for a lesson and they end up in ‘behaviour’. The two don’t often, don’t always, meet. (G1, P1)

It was also considered essential to understand what skills, provisions and support schools have already as a means of identifying strengths and acknowledging existing good work. Among areas schools could capture through this process of asset mapping were existing staff training among teaching and non-teaching staff, particularly those in pastoral roles; in-house specialist support, i.e. school counselling; any classroom teaching in operation and also the location of external mental health services relative to the school and any existing relationships with them.

#### Needs mapping and data-led action planning

There was widespread consensus that while schools may be acting on the same WSA guidance, their responses to it must be allowed to vary according to the needs and profile of their own population, including pupils, staff and families. It was noted that schools in Wales should draw on available survey data on pupil health and well-being and also that schools may already hold data that can contribute. This not only included routinely collected data on pupil health, exclusions and behaviour management, Free School Meal rates, etc. but also accessed the qualitative knowledge of school staff to ensure that content is matched to population:

Because there’s no point going in with a set of ideas, and the school, if they don’t meet the needs of the pupils that you’ve got, because you’ve read it in a book and it’s a great way forward. (G4, P3)

Consolidation of these school-level data can lead to the development of action plans, identifying how schools intend to implement evidence-led responses to their own needs. While the specific content may differ, it was noted that all schools should aim to incorporate both universal and targeted actions, with classroom sessions for all pupils, as well as more specialist support for those with higher-level needs. Aims also included identifying opportunities for embedding mental health and emotional well-being within the curriculum to ensure more sustainable delivery and develop teacher skills. In Wales, the WSA was largely viewed as complementary to the newly introduced curriculum for Wales previously outlined.

### Potential additional barriers to effective implementation

There was a significant overlap among participants when asked to discuss possible future barriers to the implementation of a WSA in schools in Wales. Issues that were commonly considered as likely to negatively impact delivery included existing school pressures, such as lack of time and funding; access to specialist support and evidence-based resources and how to capture effectiveness.

#### Typical school pressures likely to impact a WSA

Data were collected during the COVID-19 pandemic, and, as such, this was clearly a significant strain on school function at the time. While this was referenced by some, discussion primarily focused on the WSA as a new programme of work that was expected to begin postpandemic (or at least postrestrictions). Most felt that as restrictions were lifted as school returned to previous norms, recovery from the impacts of the pandemic would likely influence school capacity to act, with the expectations of increased challenges to pupil and staff mental well-being.

In terms of ongoing routine pressures, many participants referenced the lack of time in schools in general to deliver on new programmes of work and the significant pressures already experienced by school staff who are frequently expected to support well-being needs of pupils outside of the traditional educator role:

Time and pressures and the fact that they are social services as well as teachers and public health workers and goodness knows what. (G2, P4)

It was argued that a WSA should be communicated as a long-term, system-wide change rather than another task for school staff to take on, with support and training available and realistic expectation of the extent to which staff can be expected to address mental and emotional well-being issues:

You know, I think the teachers do need training, but they don’t need to know everything and anything about that subject, they just need to know enough for day to day of if something occurs. (G3, P2)

System-wide development of skills and competencies among school staff, with embedded training and professional development, was essential for sustaining change. This was seen as empowering for school staff, as a means to more effectively support both pupils and each other. It was suggested that this include time and space for reflection, which is challenging within the current school system:

They don’t get time to go and catch up with their colleagues and have a moment to reflect. They’re front facing on all the time. And so, you know, having spaces for reflection, having spaces to kind of be curious and learn together, in and of itself, you know, promotes someone’s well-being. (P1)

Some cited their own experience of successfully delivering staff training in other areas by ‘buying out’ staff time to focus on the topic at hand:

In our experience having a day where teachers can come from school if their supply cover costs can be paid. That’s a massive help with getting them there…you know they’ve got to be released from their class. So getting them in a headspace where they can sort of move away from the business of school for a minute and just focus on emotional well-being. (P2)

Securing funding for such activities and for ongoing staff development was seen as a constant challenge, increasing the temptation to look for quicker solutions:

Sometimes people are driven to whatever’s quickly and immediately and freely available. Nobody’s got an money to buy resources. (G2, P2)

#### Access to specialist support

Most people noted that, while schools are effectively the setting for the WSA, they cannot deliver it without support from external partners at all stages, including specialist mental health support. Child and Adolescent Mental Health Services (CAMHS) were seen as a key partner as the lead statutory support available; however, some had experienced challenges in accessing this service due to lack of local capacity:

But it becomes too extreme for the school, its access to has always been the issue that schools have had, because historically we couldn’t refer to CAMHS. So we were always just saying to parents you need to go to your GP, or if it’s really bad take them to A&E, and then get them access. But basically the CAMHS system is very stretched, particularly across South Wales. (G4, P3)

The newly implemented CAMHS In-Reach to Schools Pilot Programme in Wales [27], which aims to improve access to specialist support and to increase school capacity to respond to issues internally, was referenced as a potentially positive development. However, significant variations in the availability of specialist support, including provision for Welsh speakers, were acknowledged as a challenging issue requiring additional resourcing and long-term monitoring:

The long-term aim has to be for equitable availability of services, in the language of choice of the child, you’re a long way off that in some parts of Wales and you can’t deal with that overnight. (G1, P4)

#### Capturing impact

Groups discussed how implementation and any effects of a WSA could be captured, and it was generally agreed that this must be communicated to schools as a process, with more immediate emphasis on implementation as a mechanism to attain longer-term improvements in mental and emotional well-being. Large-scale, long-term evaluation was recommended to fully capture implementation and school-level change over time.

Groups also discussed the potential for a system of accreditation for schools, including incorporation into the existing Wales Network of Healthy Schools scheme. There were mixed views on this approach. Some suggested that an element of standardization is key to ensure consistency of support across Wales, by not only defining minimum required elements of a WSA but also allowing for flexibility for schools to tailor to their own settings. Others suggested that accreditation may be problematic, implying that schools that are assessed as meeting the defined standard may then be less inclined to make further changes or improvements in the future:

The distance travelled measure is maybe more important than putting kind of labels on specific outcomes I’d say definitely. Yeah, sometimes I don’t know if outcomes are helpful because you aim for that outcome then, when you could be aiming way higher. (P1)

Furthermore, accreditation may suggest a ‘pass/fail’ standard, which some felt can be damaging and unhelpful:

When, say, a school is deemed as failing, it’s a very publicly shaming system. We get this traffic light system; we get loss of faith and collaboration from parents in the community and schools are stigmatised and regarded as failing and not very good. (G5, P2)

This must be mitigated with clear communication to schools on the WSA as a developmental, iterative process and any accreditation as part of this process.

## Discussion

This research presents stakeholder perspectives on how to initiate a WSA to mental health and emotional well-being. It provides pragmatic and valuable insights to support the rollout of the new statutory guidance ‘Framework on Embedding a Whole School Approach to Emotional and Mental Well-Being’ [16] introduced in Wales.

Data here reflected existing definitions of what a WSA is, referring to a system-wide programme of change involving all stakeholders both within and around the school [17]. While the primary focus may be on improving pupil outcomes, participants noted the importance of focusing on staff well-being as both part of WSA implementation and to underpin effective working with children and young people. This was seen as key where school staff are being asked to potentially expand their roles to support mental health and emotional well-being. There is limited clear evidence of most effective approaches to supporting staff who are themselves supporting children and young people with mental and emotional issues [28]; however, it is argued that schools should be psychologically safe spaces for teachers [29], providing opportunities to share own experiences and to learn skills to support both pupils and colleagues. Senior leadership commitment to staff well-being is widely noted as important [30], including providing staff with opportunities to receive training on working with mental and emotional well-being issues and incorporating this into recognized Continuing Professional Development [21]. An absence of staff training can undermine implementation of whole school programmes [20]. However, capacity to focus on staff well-being is likely to be impacted by existing pressures of workload, staffing levels, etc., some of which will not be amenable to change by individuals within schools. Here, participants noted that staff training must also be supported by resources, in terms of both time to commit to any such activities and also financial support to ensure that schools can provide cover for staff time.

This highlights a recurring theme of existing pressures within the school system, which may act to undermine any shift to working in more recognizably ‘whole school’ ways. Participants emphasized the lack of time for staff to add further duties to their roles, which are already multifaceted and extended beyond traditional pedagogical activities. While these were noted here as a normal part of school life, these issues were perhaps brought into focus even more during the COVID-19 pandemic, where workloads were being reported as unmanageable by significant numbers of school staff [31], and there has been a marked deterioration in teacher mental health and well-being [32]. The new statutory guidance for Wales is being introduced at a time of significant pressures and risks being seen as additional work by those who are pivotal to effectiveness. Evidence suggests that school staff are more supportive of whole school programmes where they are involved in early programme planning [20] and where they feel capable of continuing their own skills to delivery instead of receiving more prescriptive guidance [33], suggesting the value of initial engagement activities with school staff at an early stage of programme delivery.

Interviewees also discussed the importance of reviewing existing school policies as part of understanding existing practice, including ensuring consistency between health and behavioural strategies. At the level of individual schools, implementation of a WSA is less likely to be effective where not supported by clear internal policy and guidance available to those within the school setting [34]. While participants here noted the importance of all stakeholders being involved in the delivery of a WSA, evidence suggests that clear communication of a whole school vision, which identifies the overall programme aims, should be led by school leaders [35]. This not only includes direct action to improve student outcomes but also requires actions to support and empower other staff to generate change [36]. While school leaders can feel constrained in making system changes by the accountability-driven culture in which they operate [37], they may be supported in this through professional networks, providing safe spaces to explore and collaborate with other senior leaders on desired (or required) changes [38].

Communication may benefit from formalization into a protocol for message content and communication [39], which guides discussions on a WSA with those in and around the school system, i.e. parents/carers, pupils and specialist practitioners. This consistent messaging can support stakeholder engagement, here highlighted by participants as key to supporting WSA development and implementation. Participants felt that this school-level policy should be supported by consistent policy and guidance from the Welsh Government, communicating the aims of a WSA as well as guidance on implementation and capturing outcomes. Better understanding of the WSA programme among stakeholders is associated with more effective implementation, through increasing buy-in to programme aims [33] and increasing acceptability and engagement [31], again stressing the importance of communication at the local, as well as national, level in WSA delivery.

Effective implementation of WSA programmes involves understanding how planned activities intersect with contextual factors [40], including existing school activities on mental and emotional well-being. Here, participants highlighted variations in school contexts as likely to be significant in multiple ways to the delivery of a WSA, suggesting that these be captured in reviewing existing practice and mapping needs to understand more about existing activities and school populations. Schools in Wales are starting from different points, with some already adopting recognizably ‘whole school’ approaches to pupil well-being [41], meaning that adoption of any new programme is less likely to be distinguishable from usual practice in these settings. New programmes are always delivered within a context of existing activity, which they will interact with and, in some cases, displace, [42] meaning that an understanding of existing activity is key to assessing how a programme is implemented [23] and what outcomes are associated with the new programme rather than existing approaches. Here, participants had mixed views on capturing implementation by measuring a set of standardized activities that may represent ‘minimum’ actions for a WSA. While some felt that this was important for consistent delivery of the approach and equity of support provision for pupils, concerns were also expressed over this becoming something that schools can pass or fail. Furthermore, where school staff do not feel supported by funding and training to implement mandated reforms, effectiveness and likelihood of change are lessened [43].

Capturing and recognizing existing practices is also associated with greater acceptability for new approaches among stakeholders [44] and is a means to acknowledge existing good work. Furthermore, without an understanding of current practices to support mental health, understanding the degree of change occurring as a result of a new programme is unlikely. While acknowledging that the result of these mapping activities—and subsequent school responses to identified need—will vary, it may be desirable for the process of capturing existing activities and population needs to be a standardized component of WSA implementation. To this end, the Welsh Government recommends [16] that secondary schools in Wales draw on data from the biennial health and well-being survey administered through the School Health Research Network (see https://www.shrn.org.uk/). This provides school population data that can support action planning; however, there are limitations to the extent to which schools can identify more at-risk groups and those with higher support needs, which may require more detailed exploration within schools. Furthermore, while data on needs may be available to schools, the capacity to act on this need depends on multiple factors, including staff time, resources and access to evidence-led intervention.

Participants also stressed variations in access across Wales to specialist mental health support for those pupils with higher-level needs that may require more intervention. This was particularly noted in relation to CAMHS as well as access to educational psychologists, where variations in availability across Wales were noted. Evidence suggests historical difficulties for many young people in accessing CAMHS, associated with lack of appropriate referrals by adults in their lives, long waiting times and lack of available local services [41]. While the WSA in Wales may be primarily a school-based programme, it incorporates working with specialist services, and therefore, the availability of these services in all areas is key to both delivery and health equity, through ensuring consistent access to support. The recent CAMHS In-Reach to Schools Pilot Programme in Wales [27] involved CAMHS practitioners working with school staff to increase confidence in recognizing and responding to mental health concerns in pupils, as well as improving access to specialist support. This programme is being supported by the Welsh Government, will be rolled out across Wales and may be influential in supporting school delivery of a WSA. Any evaluation of the framework in Wales should also incorporate exploration of other system changes such as this to identify improvements to support available to schools.

Evidence from existing programmes supports associations between quality of implementation and programme outcomes [22], with historical concerns over a lack of reporting of implementation processes in studies of school-based mental health programmes [42]. Participants here recognized the challenges in assessing effectiveness of a WSA, due to the potential timescales of implementation and the variations in populations across schools. Coupled with an absence of long-term follow-up data from whole school mental health programmes [20], this suggests that any evaluation of the statutory guidance for Wales should incorporate a focus on implementation processes along with the collection of longer-term outcomes data on mental and emotional well-being.

## Conclusion

At the time of writing, the Framework Guidance is now in use within schools in Wales, with implementation being supported by Public Health Wales through Welsh Government funding. This research is therefore timely in supporting evaluation activities going forward.

Understanding how schools use the Framework Guidance, including capture or displacement of existing practices as well as changes to the school system, will facilitate interpretation of any subsequent findings on pupil and staff mental well-being. It will also support understanding of how the programme may be subject to local adaptations over time due to contextual factors and the effects of any such changes. The author team are leading an evaluation of the rollout and impact of the Framework over the next several years, which will aim to capture data on pupil mental health and well-being, school-level changes to practices and system-wide changes in those services supporting schools. This will be a mixed-methods evaluation with publications forthcoming on interim and final results.

## Limitations

This paper describes small-scale qualitative research with purposive sampling to identify a range of stakeholders with the capacity to provide significant insights into the research problem. While population representativeness is not the aim of such research [43, 44], it is possible that other stakeholders may have differed in their views of the issue.

While online data collection may have benefits in terms of accessibility and time saved [24], as well as providing increased comfort for participants in their own surroundings [45], there may also be limitations for such approaches. These can include loss of nonverbal communications and access issues related to technology [46]. The absence of cues for interviewers to expand on areas of discussion or bring others into conversations may also reduce nuance [44]. While acknowledging these potential limitations and the circumstances that dictated data collection methods, in this case, all research staff were experienced in conducting interviews, resulting in rich data for analysis.
